# Effects of insulin signaling on mouse taste cell proliferation

**DOI:** 10.1371/journal.pone.0225190

**Published:** 2019-11-12

**Authors:** Shingo Takai, Yu Watanabe, Keisuke Sanematsu, Ryusuke Yoshida, Robert F. Margolskee, Peihua Jiang, Ikiru Atsuta, Kiyoshi Koyano, Yuzo Ninomiya, Noriatsu Shigemura

**Affiliations:** 1 Section of Oral Neuroscience, Faculty of Dental Science, Kyushu University, Fukuoka, Japan; 2 Monell Chemical Senses Center, Philadelphia, PA, United States of America; 3 Section of Removable Prosthodontics, Division of Oral Rehabilitation, Faculty of Dental Science, Kyushu University, Fukuoka, Japan; 4 Department of Oral Physiology, Graduate School of Medicine, Dentistry and Pharmaceutical Sciences, Okayama University, Okayama, Japan; 5 Division of Sensory Physiology, Research and Development Center for Five-Sense Devices Taste and Odor Sensing, Kyushu University, Fukuoka, Japan; The University of Tokyo, JAPAN

## Abstract

Expression of insulin and its receptor (IR) in rodent taste cells has been proposed, but exactly which types of taste cells express IR and the function of insulin signaling in taste organ have yet to be determined. In this study, we analyzed expression of IR mRNA and protein in mouse taste bud cells in vivo and explored its function ex vivo in organoids, using RT-PCR, immunohistochemistry, and quantitative PCR. In mouse taste tissue, IR was expressed broadly in taste buds, including in type II and III taste cells. With using 3-D taste bud organoids, we found insulin in the culture medium significantly decreased the number of taste cell and mRNA expression levels of many taste cell genes, including nucleoside triphosphate diphosphohydrolase-2 (NTPDase2), Tas1R3 (T1R3), gustducin, carbonic anhydrase 4 (CA4), glucose transporter-8 (GLUT8), and sodium-glucose cotransporter-1 (SGLT1) in a concentration-dependent manner. Rapamycin, an inhibitor of mechanistic target of rapamycin (mTOR) signaling, diminished insulin’s effects and increase taste cell generation. Altogether, circulating insulin might be an important regulator of taste cell growth and/or proliferation via activation of the mTOR pathway.

## Introduction

Insulin is an essential hormone for managing energy within the body. It is released from pancreatic islet β-cells in response to blood glucose rise, facilitates the transfer of glucose transporters to the membrane, promotes absorption of glucose into fat and skeletal muscle cells, and inhibits hepatic glucose production. In rodent taste cells, expression of insulin and the insulin receptor subunit α (IRα) has been proposed [1], [2]. In mouse circumvallate papillae (CV), evidence of insulin signaling was found along with 1-phosphatidylinositol-4,5-bisphosphate phosphodiesterase beta-2 (PLCβ2) or synaptosome-associated protein 25 (SNAP25) [2]. In mature rat taste cells, some IRα-positive taste bud cells expressed keratin 18, a type I cytokeratin [3]. In addition, electrophysiological experiments with isolated mouse taste cells demonstrated that insulin may influence salt taste sensitivity by controlling the open probability of epithelial sodium channel (ENaC) and transport of ENaC proteins to the membrane [4], [5], suggesting a subpopulation of ENaC-expressing type I taste cells might be insulin sensitive [6]. However, exactly which types of mouse taste cells express IR remains to be determined, and little is known about its function in the peripheral taste system.

Many studies note the contribution of insulin to cell growth and differentiation, indicating fundamental roles of insulin in overall cell physiology [7]. Insulin could activate the mechanistic target of rapamycin (mTOR), a serine-threonine kinase that is a key molecule for the regulation of cell growth, protein synthesis, and autophagy, depending on the availability of nutrients, growth factors, and energy [8], [9], [10], [11]. In cultured pancreatic αTC1 cells, insulin treatment up-regulated the mTOR signaling pathway and increased insulin-mediated proliferation in a concentration-dependent manner [12]. It is possible that insulin signaling and consequent mTOR activation could be involved in taste cell differentiation or proliferation.

Taste cells are continuously renewed throughout life, with an average life span of 10–14 days [13], [14], [15]. Taste bud cell turnover relies on taste bud progenitor/stem cells that express Lgr5, 6, or 4 [16]. Recently, using an ex vivo 3-dimensional (3-D) taste stem cell culture system revealed that taste progenitor/stem cells can differentiate into all types of taste cells, including type I, II, and III cells [17], and several growth factors and chemical mediators are known to contribute to maintaining functional/structural homeostasis of taste buds, for example, bone morphogenetic proteins [18], neurotrophin [19], fibroblast growth factors [20], insulin-like growth factor-1 (IGF1) [21], sonic hedgehog (Shh) [22], and Wnt proteins [23].

In this study, we explored the role of insulin signaling on the generation of taste cells. We first examined IR mRNA and protein expression in both anterior and posterior parts of the mouse tongue. Next, using an ex vivo three-dimensional taste stem cell culture system that generates taste bud organoids, we found that insulin in the culture medium suppressed taste cell proliferation. Further, we found that blocking mTOR drastically increased all types of taste cell generation in these organoid colonies.

## Materials and methods

### Animals

Mouse husbandry and all mouse experiments were carried out under the ethical guidelines of Kyushu University. All experimental protocols and procedures were approved by the Committee for Laboratory Animal Care and Use at Kyushu University in accordance with the National Institutes of Health *Guide for the Care and Use of Laboratory Animals* (approval no. A29-206). C57BL/6Jmice were purchased from Charles River Laboratories Japan, Yokohama, Japan (n = 57). GAD67-green fluorescent protein (GFP) mice (n = 3), on a C57BL/6J (B6) genetic background, were generated by Dr. Yuchio Yanagawa and the details were previously described in [24]. All mice were maintained in specific pathogen free on a 12/12-h light/dark cycle at 23°C and had ad libitum access to water and food pellets (CE-2, CLEA Japan, Tokyo, Japan). In all experiments, both males and females 8–12 weeks of age were used.

### Immunohistochemistry

The dissected tongues of GAD67-GFP mice (n = 3, 8–12 weeks of age, 25.7 ±1.9g) were fixed in 4% paraformaldehyde (PFA) in PBS for 50 min. After dehydration with sucrose solution (10% for 1 h, 20% for 1 h, 30% for 3 h at 4°C), the frozen block of fixed tissue was embedded in Optimal Cutting Temperature (OCT) compound (Sakura Finetek, Tokyo, Japan) and sectioned into 10-μm-thick slices, which were mounted on silane-coated glass slides. Next, sections of the tongue were incubated in Histo VT One (Nakalai Tesque, Kyoto, Japan) for 20 min at 80°C for antigen retrieval and then incubated for 1 h in Blocking One solution (Nakalai Tesque). Sections were then incubated overnight at 4°C with the primary antibodies. After washing with TNT buffer, the slides were incubated in secondary antibodies for 2 h and then washed again. For taste bud organoids, we used the whole-mount immunostaining preparation as described in [17]. Briefly, the colonies from each well were collected in 1.5 ml Eppendorf tubes, washed with PBS, and fixed with 4% PFA in PBS for 15 min at room temperature. Then they were washed with TNT buffer and incubated in Blocking One solution for 1 h. Next, they were incubated with primary and secondary antibodies overnight and for 2 h, respectively.

Immunofluorescence of labeled taste cells was observed using a laser scanning microscope (FV-1000, Olympus); images were obtained using Fluoview software (Olympus, Japan). To determine the number of cells expressing GFP, T1R3, and IR, we counted positive cells in each taste bud in horizontal sections of CV or organoids. Image-ProPlus (ver. 4.0; Mediacybernetics, MD, USA) was used to exclude artifactual signals; the cells showing a signal density greater than the mean plus two standard deviations of the density in taste cells in the negative control (primary antibodies omitted) were considered positive.

The primary antibodies used in this study were anti-GFP (1:1000; chicken anti-GFP, cat. no. GFP-1020, Aves Labs, Inc., OR, USA), anti-T1R3 (1:100; goat anti-T1R3, cat. no. sc-22458, Santa Cruz Biotechnology, TX, USA), anti-CA4 (1:100; goat anti-CA4, cat. no. AF2414, R&D Systems, MN, USA), anti-IR (1:100; rabbit anti-IR, cat. no. ab203746, Abcam, Cambridge, UK), anti-Lgr5 (1:100; rat anti-mouse Lgr5, cat. no. MAB8240, R&D Systems), and anti-mTOR (1:100; mouse anti-mTOR cat. no. 215Q18, Thermo Fisher Scientific, MA, USA). The secondary antibodies used were for GFP (1:300; CF^™^543 donkey anti-chicken IgY, cat. no. 20310–1, Biotium, CA, USA), T1R3 and CA4 (1:300; Alexa Fluor 488 or 568 donkey anti-goat IgG, cat. no. A11055 or A11057, Invitrogen, OR, USA), mTOR (1:300; Alexa Fluor 488 donkey anti-mouse IgG, cat. no. A21202, Invitrogen), Lgr5 (1:300; Alexa Fluor 647 donkey anti-rat IgG, cat. no. A31571, Invitrogen), and IR (1:500; cat. no. 80067, peroxidase-conjugated AffiniPure donkey anti-rabbit IgG, Jackson Immuno Research Laboratories, PA, USA). IR was detected with tyramide signal amplification kit Alexa 647 or 568 (cat. no. T20926 or B40956, Thermo Fisher Scientific).

### Taste bud organoids

3-D taste bud organoids were prepared as previously described [25]. Briefly, the trypsinized and filtered CV tissue of C57BL/6Jmice (8–12 weeks of age, 25.1 ±1.8g, 9 mice for one preparation) was cultured in a 24-well ultra-low-attachment dish (cat. no. CLS3473, Corning, NY, USA) with CM (500 μl/well). To assess the effect of insulin, we used the following insulin-free medium: 20% DMEM/F12 medium (cat. no. 11320033, Life Technologies, OR, USA), 50% Wnt3a CM (generated from a Wnt3a-producing cell line, gift from Dr. Hans Clevers, selected by 125 μg/ml Zeocin in DMEM/F12 medium), 20% R-spondin CM (generated from an R-spondin cell line, a gift of Dr. Jeffery Wittsett, selected by 600 μg/ml Zeocin in DMEM/F12 medium), and 10% Noggin CM (generated from pEAK-Rapid cell line, selected by 400 μg/ ml Zeocin in DMEM/F12 medium), supplemented with EGF (50 ng/ml; cat. no. 315–09, Peprotech, NJ, USA), N21-MAX insulin-free media supplement (2% vol/vol; cat. no. AR010, R&D Systems), B-27^™^ Supplement minus insulin (2% vol/vol; cat. no. A1895601, Life Technologies), and penicillin-streptomycin (1×; cat. no. 15140122, Thermo Fisher Scientific) plus 5% chilled Matrigel (cat. no. 356231, Corning). For the freshly dissociated single CV cells, Y-27632 (10 μM; cat. no. Y0503, Sigma-Aldrich, MO, USA) was added in the medium to prevent dissociation-induced apoptosis. Insulin (0–50 nM; cat. no. 093–06471, Wako, Japan) was added into the CM. The concentration of insulin in CM was quantified using an insulin ELISA kit (cat. no. 10-1113-01, Mercobia, Sweden). The insulin content of all the CM that we used in this study was lower than the detection limit of this ELISA kit (3 mU/l). The CM was renewed daily from day 7 to day 20. Bright-field images of organoids were obtained by CKX-41 (Olympus) and processed with Image J software.

### PCR

To collect mouse taste tissue, animals (C57BL/6Jmice, n = 3, 8–12 weeks of age, 25.9 ±2.5g) were anesthetized with isoflurane and euthanized by cervical dislocation. The epithelia of the anterior and the posterior parts of the tongue were peeled away after elastase injection (0.5–1 mg/ml; Elastin Products, Owensville, MO, USA; incubated for 10 min at room temperature). After peeling off and removing surrounding tissue, taste buds in FP (50 taste buds per one mouse) were collected in Tyrode solution (in mM: NaCl 140, KCl 5, CaCl_2_ 1, MgCl_2_ 1, NaHCO_3_ 5, glucose 10, sodium pyruvate 10, HEPES 10; pH 7.4 adjusted with NaOH) with a glass pipette. CV tissue was dissected from surrounding tissue and Von Ebner's glands were removed under a microscope. The RNAs of mouse taste tissue or organoids were extracted with FastGene^™^ RNA Premium Kit (cat. no. FG-81050, Nippon Genetics, Japan) and assessed at a ratio of A260/A280 using LSM NanoDrop ND-1000 (Thermo Fisher Scientific). SuperScript VILO Master Mix (cat. no. 11755050, Thermo Fisher Scientific) was used for cDNA synthesis. PCR was performed as follows: 15 min 95°C (1 cycle); 30 s 94°C, 30 s 58°C, 60 s 68°C (35 cycles); 5 min 75°C (1 cycle). Each 20-μl PCR solution contained 0.5 U Taq DNA polymerase (TaKaRa Ex Taq HS, Takara, Shiga, Japan) and 2 μl 10× PCR buffer containing 20 mM Mg^2+^, 0.6 mM of each primer pair, 0.2 mM of each dNTP, and 0.4 μl cDNA solution. The amplified products were visualized using gel electrophoresis (2% agarose with GelRed Nucleic Acid Gel Stain, cat. no. 41001-41003-T, Biotium) under UV illumination. To control for signals from genomic DNA, purified RNA samples were treated in parallel with or without reverse transcriptase. For quantitative real-time PCR, Fast SYBR Green Master Mix (cat. no. 4385612, Fisher Scientific) was used. Data were analyzed with StepOne Software (ver. 2.3, Applied Biosystems, CA, USA). Each assay was performed in duplicate, and the runs were repeated three times. All qPCR results were normalized using the ΔΔCt method with *Gapdh* in each sample as reference. [26]. All primer pairs were chosen such that each primer was from a different exon. The primers used for each gene are indicated in Table 1.

**Table 1:** Primers for PCR.

	Forward primer	Reverse primer	product size
NTPDase2	ATGGCTGGAAAGTTGGTGTCA	TCTTGGGTAGGGACGCACA	92
T1R3	CAAGGCCTGCAGTGCACAA	AGGCCTTAGGTGGGCATAATAGGA	92
Gust	AGGGCATCTGAATACCAGCTCAA	CTGATCTCTGGCCACCTACATCAA	196
CA4	TTGTCACTGCTAGGACAAAGGTGAA	TCCGATAATGCACGCACCTC	140
krt8	TGAACAACAAGTTCGCCTCCTT	GCTCCTCGACGTCTTCTGCT	110
IR	CAGGAATGGCTTGTTTGGTCTTTA	TGCAGTGCTATGGCACATTTGA	123
SGLT1	TCAGGCTCAGGTGGACTTTGTG	ATCATTGGCCATGCAATGAAAC	127
GLUT4	CTGTAACTTCATTGTCGGCATGG	AGGCAGCTGAGATCTGGTCAAAC	161
GLUT8	TCTGCATGTCAAGGGTGTGG	AGGGACAACGGTCAGTGTGAATAG	175
mTOR	CTTGGAGAACCAGCCCATAA	CTGGTTTCACCAAACCGTCT	85
lgr5	TAAAGACGACGGCAACAGTG	GATTCGGATCAGCCAGCTAC	199
Gapdh	TGTGTCCGTCGTGGATCTGA	TTGCTGTTGAAGTCGCAGGAG	150

### Statistical analysis

Statistical analyses, including calculation of mean and standard errors, were performed using the statistical software packages IBM SPSS Statistics (IBM, NY, USA). One-way ANOVA and post hoc Tukey HDS tests were used to evaluate the qPCR data for each insulin concentration. Unpaired t-tests were used to evaluate rapamycin’s effects on organoids. p-Values < 0.05 were considered significant.

## Results

### Insulin receptor is expressed in taste cells

**Fig 1:**
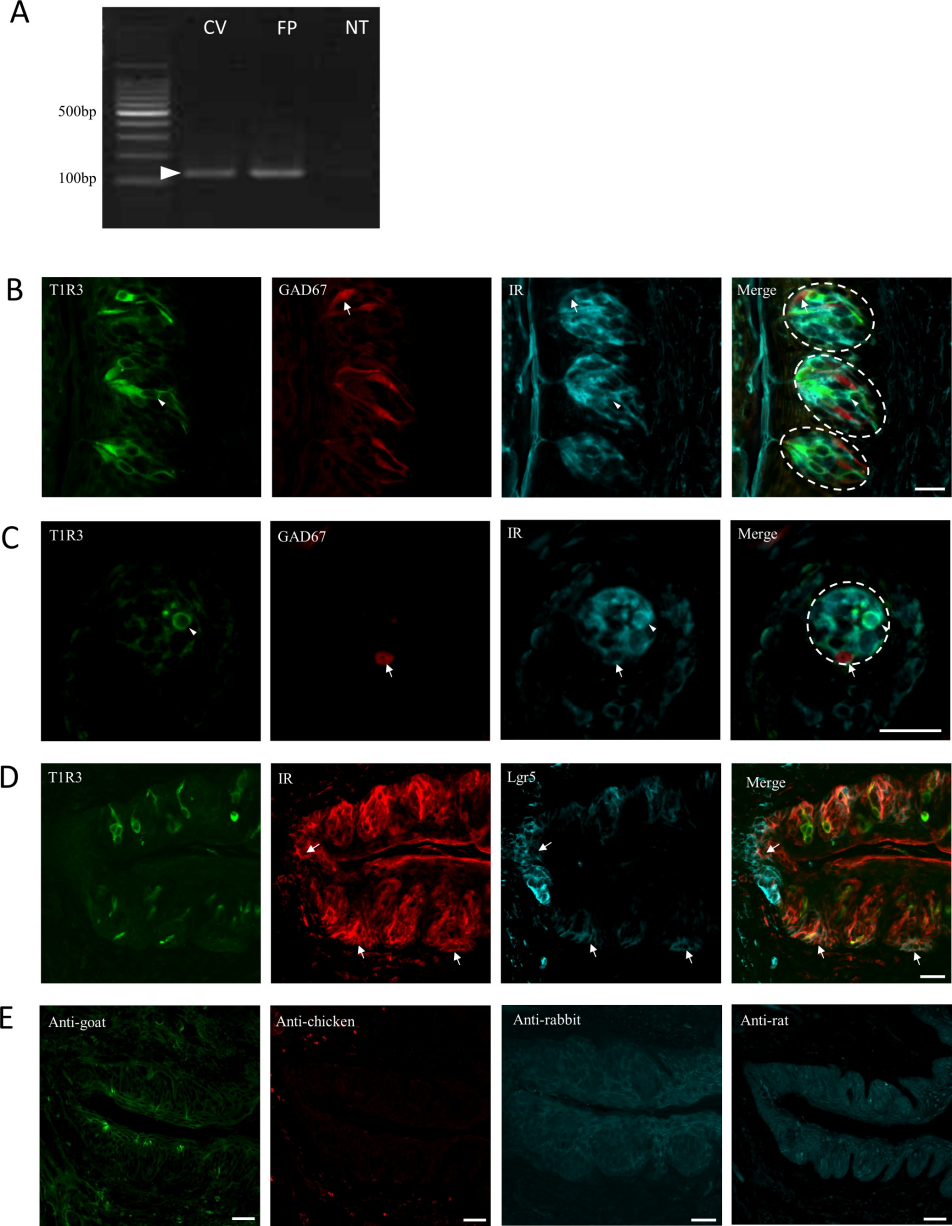
Insulin receptor (IR) mRNA and protein expression in mouse taste tissue. (A) RT-PCR showed IR mRNA expression in circumvallate (CV) and fungiform (FP) tissues but only very weak expression in non-taste epithelium (NT). (B, C) IR immunoreactivity (cyan) was observed in both CV and FP taste bud cells. Many IR+ cells were also T1R3+ (green) or GAD67+ (red). Some of the intragemmal cells negative for T1R3 and GAD67 were IR+. Representative confocal images are shown of CV (B) and FP (C). Arrows and arrowheads denote IR+GAD67 double-positive cells and T1R3+IR double-positive cells, respectively. Scale bars: 50 μm. (D) IR signals were observed at the bottom of CV trenches and near the basolateral area, overlapping with Lgr5 signals (arrows). Scale bar: 50 μm. (E) No immunoreactivity was observed when primary antibodies were omitted. Scale bar: 20 μm. Three different GAD67-GFP mice were used for the analyses.

RT-PCR indicated that IR mRNA was expressed in both CV and FP tissues, and weak expression was observed in non-taste epithelium (NT) (Fig 1A). Immunohistochemistry showed that IR immunoreactivity was found broadly among the taste cells: ~80% of T1R3-positive type II taste cells and ~60% of GAD67-GFP positive type III cells expressed IR in CV and FP; the co-expression ratios of IR and T1R3 or GAD67 were, in CV, T1R3+IR/IR = 225/281 (80.1%) and GAD67+IR/IR = 111/159 (69.8%); in FP, T1R3+IR/IR = 73/88 (83.0%) and GAD67+IR/IR = 18/30 (60.0%) (Fig 1B and 1C). IR signals were also observed in many taste bud cells that did not express either T1R3 or GAD67-GFP; thus, IR might be expressed not only in the T1R3+ and GAD67+ taste cells but also in other types of taste cells. IR immunoreactivity was also observed in Lgr5-positive taste progenitor cells (Fig 1D), suggesting that taste progenitor cells possess insulin signaling components as well. Without primary antibodies, no specific fluorescence was observed in the same samples (Fig 1E).

### Insulin effects on expression of taste cell markers and organoid colony size

**Fig 2:**
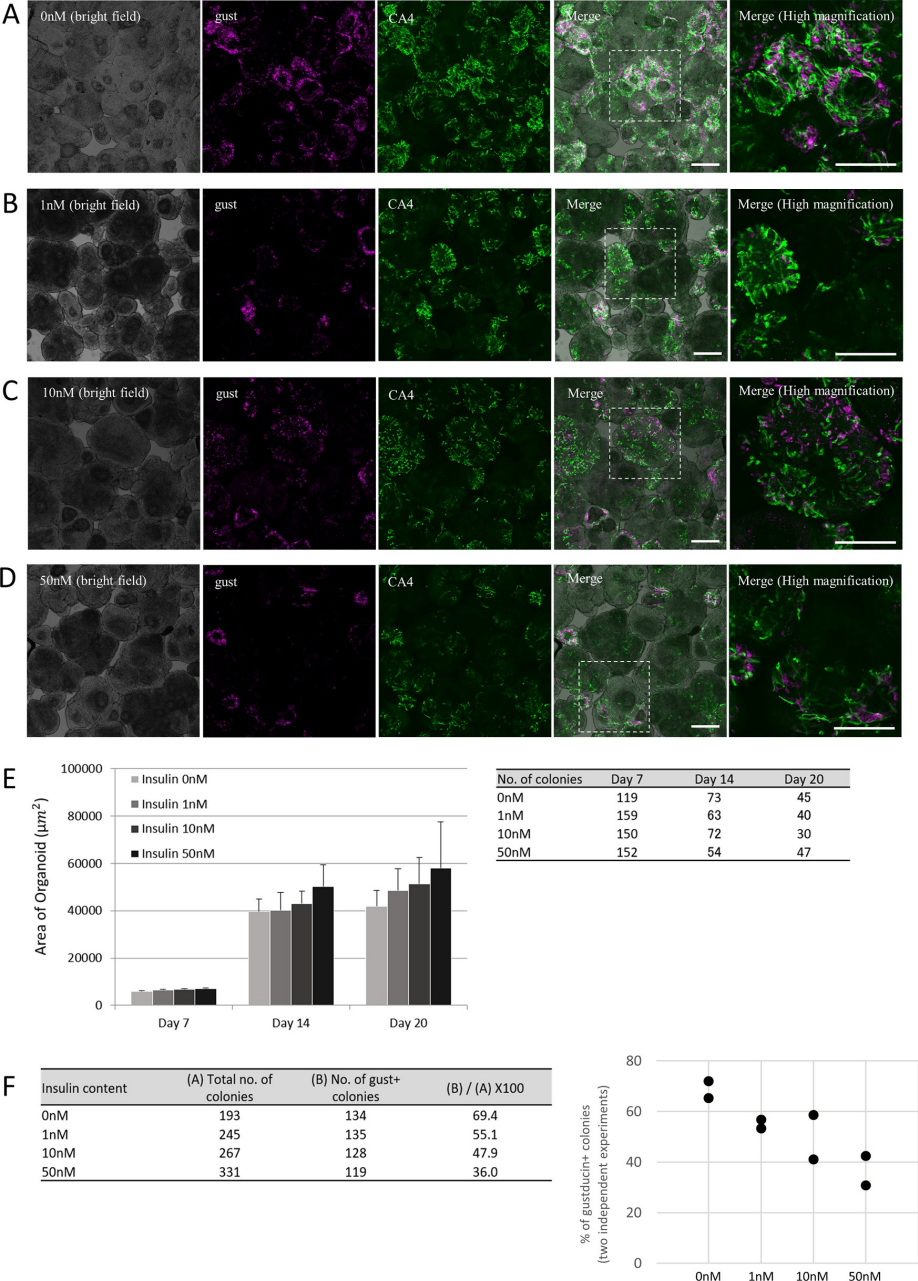
Insulin effects on taste cell generation in organoids. (A-D) Representative confocal images of taste bud organoids prepared from mouse circumvallate (CV) tissue grown in conditioned medium (CM) with varying insulin concentrations: (A) 0 nM, (B) 1 nM, (C) 10 nM, and (D) 50 nM. Each organoid was immunostained at day 20 with anti-gustducin (magenta: pseudocolor) and anti-CA4 (green) antibodies. Scale bars: 50 μm. (E) The size of organoid colonies for each insulin concentration was measured at days 7, 14, and 20. The number of colonies analyzed is indicated in the chart to the right. Data are mean + SEM. (F) The percentage of colonies that contained gustducin decreased as insulin increased. Data were collected from two independent preparations.

**Fig 3:**
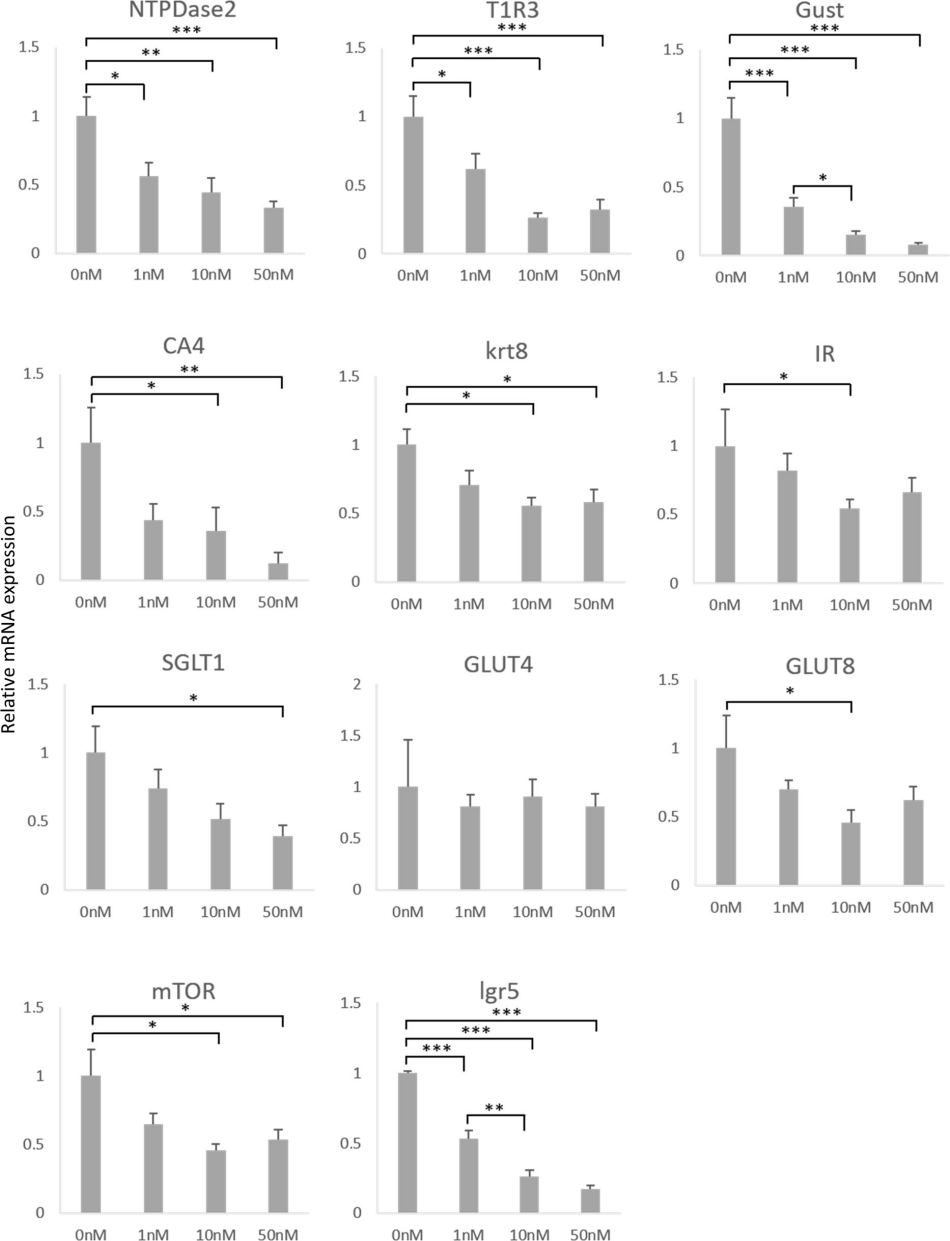
Insulin effects on taste cell type marker mRNA in taste organoids. qPCR determined the relative mRNA expression levels of the taste cell type markers indicated. Markers for type I (NTPDase2), type II (T1R3 and gustducin), and type III (CA4) decreased in taste organoids cultured in conditioned medium with higher added insulin. The marker for taste progenitor cells lgr5 also decreased by insulin. Data were collected from six independent preparations. Each score is expressed as mean + SEM. *P<0.05, **P<0.01, ***P<0.001, in post hoc Tukey HDS test.

To investigate the effect of insulin on taste cell growth, we applied various concentrations of insulin to isolated taste progenitor cells. Because the progenitor cells are located mainly at the bottom of the trenches of CV papillae, we collected CV tissue and dissociated them into single cells and then cultured them in the specially prepared CM containing Matrigel, which enabled 3-D colony formation. Most of organoid colonies contained functional taste cells [17], including type II gustducin-positive cells and type III CA4-positive cells like real taste bud in insulin-free CM(Fig 2A), but the few did not contained taste cells. The average size of colonies increased as the insulin concentration increased, but the size variation was large and differences were not statistically significant on day 20 (Fig 2E). On the other hand, the number of organoid colonies that contained gustducin-positive taste cells decreased as insulin concentration increased (Fig 2A–2D and 2F). Expression levels of mRNAs for each taste cell marker tested for all subtypes of taste cells (NTPDase2, type I cells; T1R3 and gustducin, type II cells; CA4, type III taste cells; krt8, mature taste cells) decreased significantly in an insulin-concentration-dependent manner (Fig 3). The mRNA expression level of lgr5, a marker molecule of taste progenitor cells, also decreased by insulin application (Fig 3). The lgr5-positive taste progenitor cells have the capability to differentiate into all types of taste cells [17], hence insulin might influence the differentiation or proliferation processes of all types of taste cells.

### mTOR inhibition increases expression of certain taste cell markers

**Fig 4:**
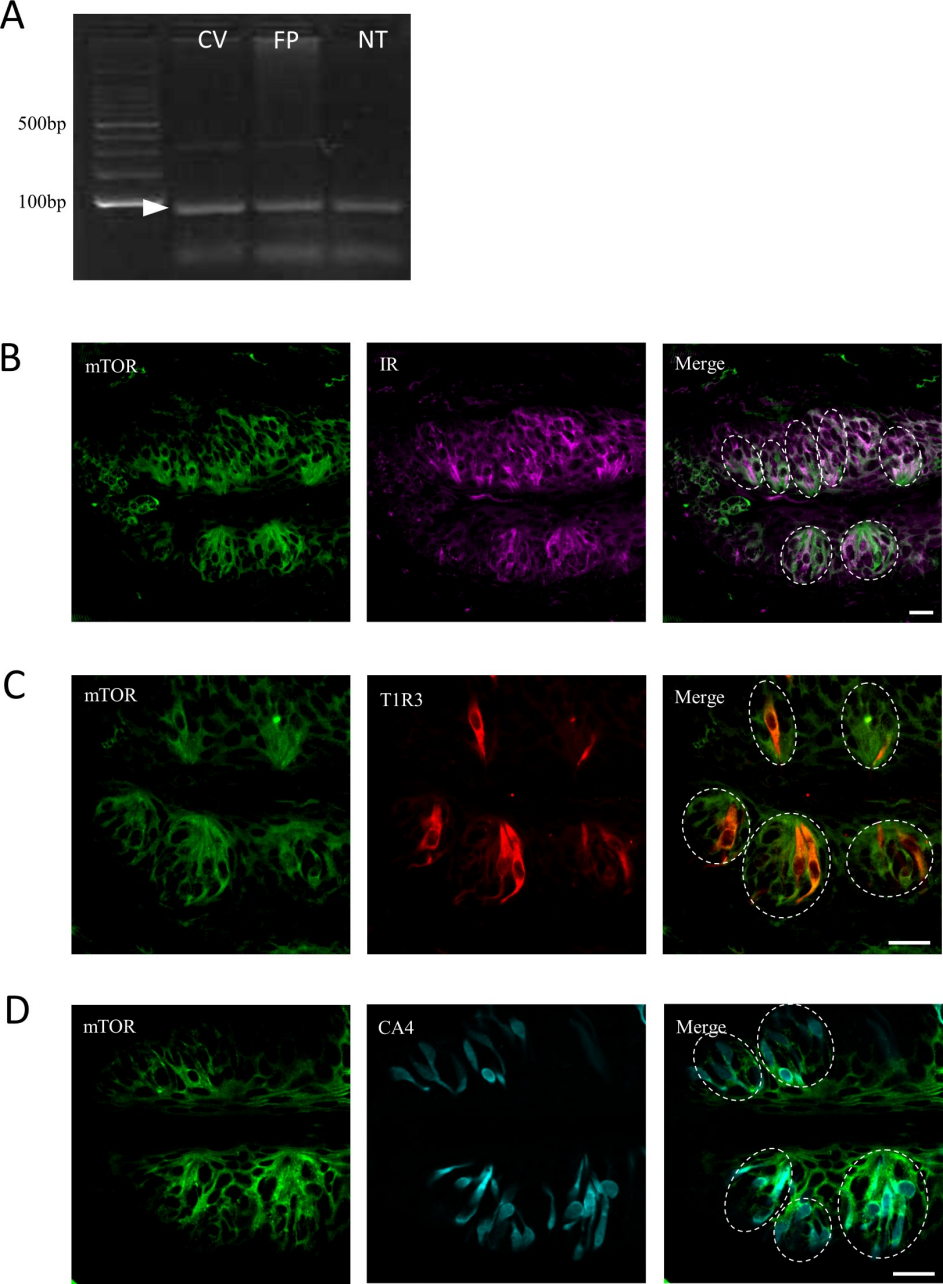
mTOR expression in mouse taste tissue. (A) mTOR mRNA expression in circumvallate (CV) and fungiform (FP) taste bud cells and non-taste tongue epithelium (NT). (B-D) mTOR immunoreactivity was observed in mouse CV taste bud cells, including many intragemmal cells which immunopositive for IR (B), T1R3 (C) and CA4 (D). Scale bar: 20 μm.

**Fig 5:**
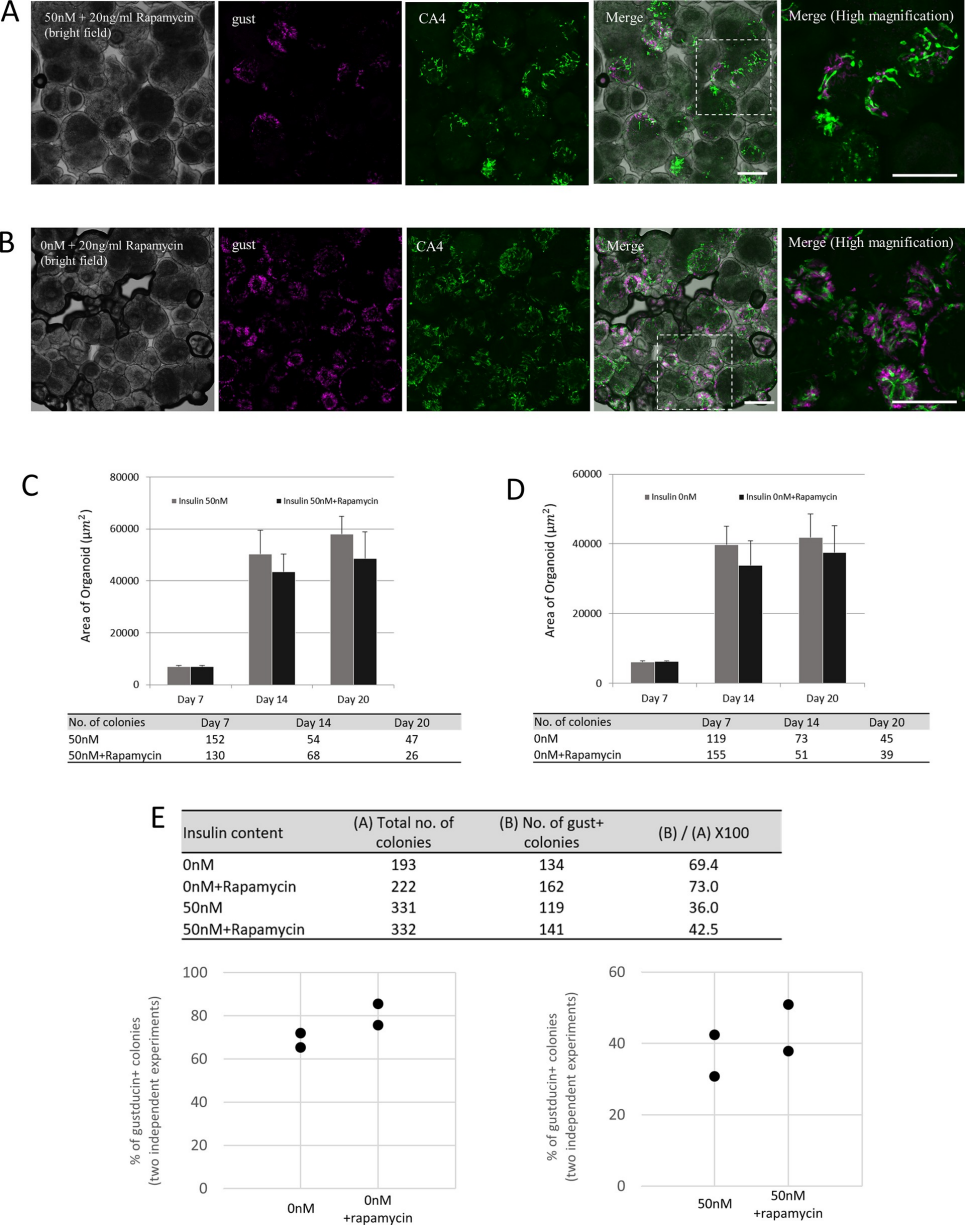
Effects of treatment with mTORC1 blocker rapamycin on taste cells. (A, B) Confocal images of taste organoids at day 20, cultured in conditioned medium (CM) with 20 ng/ml rapamycin plus 50 nM insulin (A) or 0 nM insulin (B). Scale bars: 50 μm. (C, D) Average colony size was decreased both with (C) and without (D) insulin in CM containing rapamycin (no significant difference). (E) Colonies containing gustducin-positive cells were more frequent in rapamycin treatment groups. Data were collected from two independent preparations.

**Fig 6:**
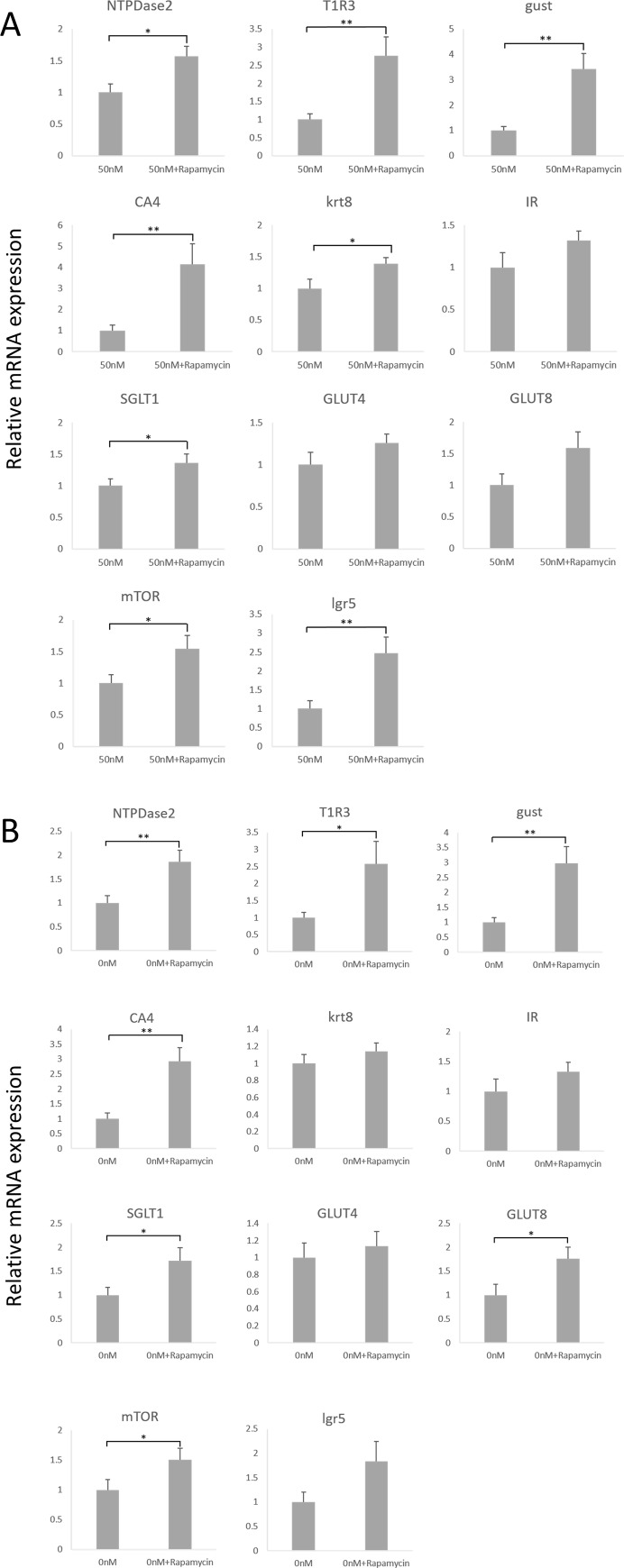
Rapamycin effects on taste cell marker mRNAs. Relative mRNA expression of taste cell markers in organoids treated with or without 20 ng/ml rapamycin. Both in 50 nM insulin conditioned medium (CM) (A) and 0 nM insulin CM (B), the mRNA expression level of many molecules which expressed in taste buds were significantly increased in the presence of rapamycin. Data were collected from six independent preparations. Scores are expressed as mean + SEM. Unpaired t-test: *P<0.05, **P<0.01.

The mTOR pathway is activated by a variety of divergent growth factors, including insulin (reviewed in [11]). By RT-PCR, mTOR mRNA was detected in CV, FP, and NT (Fig 4A). By immunostaining, mTOR protein was expressed in CV taste bud cells, including IR+, T1R3+ and CA4+ cells (Fig 4B–4D). To investigate the role of mTOR in taste cells, we added 20 ng/ml rapamycin, an mTOR inhibitor, to CM and cultured taste organoids for 20 days. Rapamycin blocks mTOR complex 1 (mTORC1), which is the complex of mTOR with Raptor [11]. Regardless of insulin content, rapamycin treatment decreased the average size of organoid colonies (Fig 5A–5D) and increased the number of the colonies containing gustducin+ taste cells (Fig 5A, 5B and 5E). Rapamycin treatment increased mRNA expression levels of taste cell markers in CM with or without 50 nM insulin (Fig 6A and 6B). In 50nM insulin CM, the statistical differences were observed in NTPDase2 (t = 2.86, p<0.05), T1R3 (t = 3.18, p<0.01), gustducin (t = 3.84, p<0.01), CA4 (t = 3.15, p<0.01), krt8 (t = 2.34, p<0.05), SGLT1 (t = 2.04, p<0.05), and mTOR (t = 2.20, p<0.05). And in 0nM CM, NTPDase2 (t = 3.06, p<0.01), T1R3 (t = 2.35, p<0.05), gustducin (t = 3.34, p<0.01), CA4 (t = 3.86, p<0.01), SGLT1 (t = 2.25, p<0.05), GLUT8 (t = 2.34, p<0.05), and mTOR (t = 2.00, p<0.05) were increased significantly. The lgr5 mRNA expression was also increased by rapamycin application, significantly in 50nM insulin CM (t = 3.03, p<0.01). These results indicate that the mTOR pathway might regulate taste cell growth and/or differentiation.

## Discussion

In the present study, we showed IR mRNA and protein expression in mouse taste organ. In taste bud organoid experiments, insulin in the medium activated the mTOR signaling pathway and regulated taste cell generation. Rapamycin, an mTORC1 inhibitor, counteracted insulin’s effect and significantly promoted the expression of taste cell markers in organoid colonies.

Culturing taste bud organoids is a recent experimental technique to create stem-cell-derived 3-D cell culture, first established by Ren et al. in 2014 [17], that generates functional taste cells from lingual taste stem/progenitor cells expressing stem cell marker LGR5 or LGR6. Their basic procedure to culture taste bud organoids followed the method developed for intestinal organoids from crypts [27], [28]. The medium used in those studies contains a number of supplements, including B-27, N2, EGF, and three different CM, which were generated by culturing HEK-293 cells with heterologously expressed growth factors (Wnt3a, R-spondin, and Noggin) in the medium. Among those ingredients, several supplements (B-27 and N2) and Opti-MEM, which were used for generating R-spondin and Noggin CM in the original recipe, contain insulin far beyond physiological concentrations (much higher than the highest concentration measured by the Mercodia insulin ELISA kit: 200 mU/l), so it was impossible to evaluate the effect of insulin on taste stem cell growth using the original CM.

We prepared the modified CM with using insulin free reagents, and confirmed insulin content using ELISA quantification. The insulin ELISA kit was not able to detect insulin content in the all CM we used in this study. *In vivo*, the normal concentration of mouse blood serum insulin is around 16.4 ± 4.5 μU/ml (~0.11 ± 0.031 nM, after 8 hours of food deprivation), and even after 16 weeks of high-fat chow feeding, their serum insulin level was 170.9 ± 14.4 μU/ml (~1.19 ± 0.01 nM, after 8 hours of food deprivation) [29]. Although in many cell culture studies, a high concentration of insulin was applied to visualize its effect, for example, to detect its proliferative effect (e.g., 0–100 nM insulin was applied to the αTC1 cells (EC_50_ = 2 nM) [12]), the experimental condition in this study was more like the physiological state in terms of insulin concentration. The taste bud organoid system mimics many aspects of taste organ growth ex vivo but still could not completely reproduce taste tissue and the surrounding environment. To elucidate the relationships among various growth factors, including insulin might be the next step to advance this system.

The mRNA levels of glucose transporters shown to be expressed in taste buds, such as SGLT1 and GLUT8, were decreased by insulin application. However, no significant change was observed in GLUT4 expression (Fig 3), perhaps because GLUT4 expression is not restricted to taste cells but also is frequently expressed in NT or mesenchymal cells [16]. In our experimental setting organoid colonies were derived from mouse tissue surrounding CV, containing taste progenitor cells and NT progenitor cells, so some colonies might have contained NT tissue, which would make it difficult to see the change in GLUT4 expression specifically in taste cells.

mTOR is known to sense environmental and cellular nutrition status. Various stimulants could activate at least two mTOR complexes, mTORC1 and mTORC2, to control cell growth, proliferation, development, longevity, and autophagy [10]. Insulin is known to activate mTORC1 by controlling the signal pathways dependent on phosphatidylinositol 3-kinase (PI3K) and Ras [30]. Our data showed that both taste cells and surrounding NT tissue expressed mTOR, and IR and mTOR signals were coexpressed in many taste bud cells including T1R3 expressing and CA4 expressing cells (Fig 4). Rapamycin, an mTORC1 inhibitor, impaired insulin’s effect and strongly promoted taste cell proliferation in the organoids (Fig 5A and 5E, and Fig 6A). Even in 0 nM insulin CM, mTORC1 inhibition increased taste cell generation (Fig 5B and 5E, and Fig 6B), perhaps because mTOR signaling was activated by glucose or some amino acid in the medium. A previous study suggested that the T1R1+T1R3 umami receptor may regulate amino-acid-induced mTOR signaling [31]. Moreover, mTOR inhibition is reported to ameliorate radiation-induced salivary gland damage [32]. A rapamycin analogue induced autophagy and then suppressed an exacerbated compensatory proliferation, which allowed for improvement and reestablishment of salivary gland function [32]. Autophagy is a homeostatic process that is constitutionally active in essentially all eukaryotic tissues [33] and is required to generate new space for newly proliferated cells during unremitting cell generation, in our experiments and probably in mouse taste organs. It is possible that excessive insulin could interrupt normal cell apoptotic processes and the consequent smooth cell turnover. In this context, insulin could be one of the important regulators to maintain normal taste cell turnover.

In our immunohistochemical study, the IR signal was observed in T1R3+ and GAD67+ taste cells (Fig 1B and 1C). A previous study reported that IRα is expressed in the spindle-shaped taste cells and is coexpressed with keratin 18 in CV [1]. And a subset of ENaC-expressing salt-responsive taste cells might express IR because amiloride-sensitive salt taste responses were enhanced by insulin via controlling the open probability of ENaC and transport of ENaC proteins to the membrane [4], [5]. Type I taste cells may express ENaC [6]; thus, a subpopulation of type I taste cells might express ENaC and IR. Altogether, as our work demonstrated, IR expression in taste buds may not be restricted to specific taste cells types, and a large population of taste cells may express IR.

According to previous reports, IGF1 receptor (IGF1R), which shares a high degree of homology with IR and has low affinity for insulin, is expressed in taste bud cells, and some overlapped with keratin 18 [21], [1]. Young (30-days-old) but not adult (80-days-old) mice genetically lacking IGF1R in lingual epithelium exhibited decreased taste bud numbers [21]. In addition, IGF1R deletion did not affect taste bud size and taste cell population [21]. According to results of our qPCR experiments with taste organoids, the taste cell marker krt8 and each cell-type marker (NTPDase2, T1R3, gustducin, and CA4) were significantly decreased with higher insulin concentrations (Fig 3). Both IR and IGF1R were shown to have broad expression patterns in taste buds (Fig 1B and 1C and [1]), but their multiple specific functions have yet to be studied in various cell types and organs. It is possible that distinct functions of IR and IGF1R might exist in taste bud development.

Taste disorder has been described frequently during the course of diabetes, for example, increased recognition thresholds for glucose, NaCl [34], and sucrose [35] and impaired sweet, sour, and salt taste detection in type II diabetic patients [36]. Hyperactive mTORC1 has been observed in obesity and nutrient overload, probably due to hyperglycemia and hyperinsulinemia [7]. According to our results, the hyperinsulinemia that advances with overweight could impact the proliferation of all taste cell types and might be a potential risk factor for impairment of total taste sensitivity. mTOR might be a new pharmacological target to improve their dietary experiences.

In conclusion, we demonstrate the broad expression of IR and mTOR in mouse taste bud cells. The ex vivo taste cell culture system revealed that insulin negatively regulated taste cell generation, including type I, II, III, and taste progenitor cells, and pharmacological inhibition of mTOR significantly promoted taste cell proliferation. This is the first study to suggest that insulin might play an important role in the taste cell differentiation/proliferation, and mTOR might be a key molecule in the maintenance of taste bud homeostasis. Further investigation is required to determine the relationship between the animal’s metabolic status and insulin-mTOR signaling, especially in pathophysiological states, such as obesity or diabetes.

## Abbreviations

CA4: carbonic anhydrase 4
CM: conditioned medium
CV: circumvallate papillae
EGF: epidermal growth factor
ELISA: enzyme-linked immunosorbent assay
ENaC: epithelial sodium channel
FP: fungiform papillae
GAD67: glutamate decarboxylase 67
GFP: green fluorescent protein
GLUT4/8: glucose transporter 4/8
IGF1: insulin-like growth factor-1
IGF1R: IGF1 receptor
IR: insulin receptor
IRα: insulin receptor subunit α
krt8: keratin 8
Lgr4/5/6: leucine-rich repeat-containing G-protein coupled receptor 4/5/6
mTOR: mechanistic target of rapamycin
mTORC1/2: mTOR complex 1/2
NTPDase2: nucleoside triphosphate diphosphohydrolase 2
NT: non-taste epithelium
PFA: paraformaldehyde
PLCβ2: 1-phosphatidylinositol-4,5-bisphosphate phosphodiesterase beta-2
qPCR: quantitative real-time PCR
RT-PCR: reverse transcriptase PCR
SGLT1: sodium-glucose cotransporter 1
SNAP25: synaptosome-associated protein 25
T1R3: taste receptor type 1 member 3
TNT: Tris-NaCl-Tween buffer

## References

[1] Suzuki Y, Takeda M, Sakakura Y, Suzuki N. Distinct Expression Pattern of Insulin-Like Growth Factor Family in Rodent Taste Buds. J Comp Neurol. 2005;482: 74–84. 10.1002/cne.20379 15612015

[2] Doyle ME, Fiori JL, Gonzalez Mariscal I, Liu Q-R, Goodstein E, Yang H, et al Insulin Is Transcribed and Translated in Mammalian Taste Bud Cells. Endocrinology. 2018;159: 3331–3339. 10.1210/en.2018-00534 30060183 PMC6112595

[3] Zhang C, Cotter M, Lawton A, Oakley B, Wong L, Zeng Q. Keratin 18 is associated with a subset of older taste cells in the rat. Differentiation. Wiley/Blackwell (10.1111); 1995;59: 155–162. 10.1046/j.1432-0436.1995.5930155.x 7589899

[4] Heck GL, Mierson S, DeSimone JA. Salt taste transduction occurs through an amiloride-sensitive sodium transport pathway. Science. 1984;223: 403–5. Available: http://www.ncbi.nlm.nih.gov/pubmed/6691151 10.1126/science.6691151 6691151

[5] Baquero AF, Gilbertson TA. Insulin activates epithelial sodium channel (ENaC) via phosphoinositide 3-kinase in mammalian taste receptor cells. Am J Physiol Physiol. 2011;300: C860–C871. 10.1152/ajpcell.00318.2010 21106690 PMC3074626

[6] Vandenbeuch A, Clapp TR, Kinnamon SC. Amiloride-sensitive channels in type I fungiform taste cells in mouse. BMC Neurosci. 2008;9: 1 10.1186/1471-2202-9-1 18171468 PMC2235881

[7] Yoon M-S. The Role of Mammalian Target of Rapamycin (mTOR) in Insulin Signaling Nutrients. Multidisciplinary Digital Publishing Institute (MDPI); 2017;9 10.3390/nu9111176 29077002 PMC5707648

[8] Dazert E, Hall MN. mTOR signaling in disease. Curr Opin Cell Biol. 2011;23: 744–755. 10.1016/j.ceb.2011.09.003 21963299

[9] Avruch J, Long X, Ortiz-Vega S, Rapley J, Papageorgiou A, Dai N. Amino acid regulation of TOR complex 1. Am J Physiol Metab. 2009;296: E592–E602. 10.1152/ajpendo.90645.2008 18765678 PMC2670622

[10] Ma XM, Blenis J. Molecular mechanisms of mTOR-mediated translational control. Nat Rev Mol Cell Biol. 2009;10: 307–318. 10.1038/nrm2672 19339977

[11] Zoncu R, Efeyan A, Sabatini DM. MTOR: From growth signal integration to cancer, diabetes and ageing. Nature Reviews Molecular Cell Biology. 2011 10.1038/nrm3025 21157483 PMC3390257

[12] Liu Z, Kim W, Chen Z, Shin Y-K, Carlson OD, Fiori JL, et al Insulin and Glucagon Regulate Pancreatic α-Cell Proliferation. Vella A, editor. PLoS One. Public Library of Science; 2011;6: e16096 10.1371/journal.pone.0016096 21283589 PMC3026810

[13] Beidler LM, Smallman RL. Renewal of cells within taste buds. J Cell Biol. Rockefeller University Press; 1965;27: 263–72. 10.1083/jcb.27.2.263 5884625 PMC2106718

[14] Hamamichi R, Asano-Miyoshi M, Emori Y. Taste bud contains both short-lived and long-lived cell populations. Neuroscience. 2006;141: 2129–2138. 10.1016/j.neuroscience.2006.05.061 16843606

[15] Perea-Martinez I, Nagai T, Chaudhari N. Functional cell types in taste buds have distinct longevities. Behrens M, editor. PLoS One. 2013;8: e53399 10.1371/journal.pone.0053399 23320081 PMC3540047

[16] Yee KK, Li Y, Redding KM, Iwatsuki K, Margolskee RF, Jiang P. Lgr5-EGFP Marks Taste Bud Stem/Progenitor Cells in Posterior Tongue. Stem Cells. 2013;31: 992–1000. 10.1002/stem.1338 23377989 PMC3637415

[17] Ren W, Lewandowski BC, Watson J, Aihara E, Iwatsuki K, Bachmanov AA, et al Single Lgr5- or Lgr6-expressing taste stem/progenitor cells generate taste bud cells ex vivo. Proc Natl Acad Sci. 2014; 10.1073/pnas.1409064111 25368147 PMC4246268

[18] Zhou Y, Liu H-X, Mistretta CM. Bone morphogenetic proteins and noggin: inhibiting and inducing fungiform taste papilla development. Dev Biol. 2006;297: 198–213. 10.1016/j.ydbio.2006.05.022 16828469

[19] Liebl DJ, Mbiene J-P, Parada LF. NT4/5 Mutant Mice Have Deficiency in Gustatory Papillae and Taste Bud Formation. Dev Biol. 1999;213: 378–389. 10.1006/dbio.1999.9385 10479455

[20] Petersen CI, Jheon AH, Mostowfi P, Charles C, Ching S, Thirumangalathu S, et al FGF Signaling Regulates the Number of Posterior Taste Papillae by Controlling Progenitor Field Size. Thesleff I, editor. PLoS Genet. Public Library of Science; 2011;7: e1002098 10.1371/journal.pgen.1002098 21655085 PMC3107195

[21] Biggs BT, Tang T, Krimm RF. Insulin-like growth factors are expressed in the taste system, but do not maintain adult taste buds. PLoS One. 2016; 10.1371/journal.pone.0148315 26901525 PMC4762545

[22] Lu W-J, Mann RK, Nguyen A, Bi T, Silverstein M, Tang JY, et al Neuronal delivery of Hedgehog directs spatial patterning of taste organ regeneration. Proc Natl Acad Sci. 2018;115: E200–E209. 10.1073/pnas.1719109115 29279401 PMC5777079

[23] Iwatsuki K, Liu H-X, Gronder A, Singer MA, Lane TF, Grosschedl R, et al Wnt signaling interacts with Shh to regulate taste papilla development. Proc Natl Acad Sci. 2007;104: 2253–2258. 10.1073/pnas.0607399104 17284610 PMC1794217

[24] Tamamaki N, Yanagawa Y, Tomioka R, Miyazaki J-I, Obata K, Kaneko T. Green fluorescent protein expression and colocalization with calretinin, parvalbumin, and somatostatin in the GAD67-GFP knock-in mouse. J Comp Neurol. 2003;467: 60–79. 10.1002/cne.10905 14574680

[25] Ren W, Aihara E, Lei W, Gheewala N, Uchiyama H, Margolskee RF, et al Transcriptome analyses of taste organoids reveal multiple pathways involved in taste cell generation. Sci Rep. 2017;7: 4004 10.1038/s41598-017-04099-5 28638111 PMC5479815

[26] Pfaffl MW. A new mathematical model for relative quantification in real-time RT-PCR. Nucleic Acids Res. 2001;29: e45 Available: http://www.ncbi.nlm.nih.gov/pubmed/11328886 10.1093/nar/29.9.e45 11328886 PMC55695

[27] Sato T, Vries RG, Snippert HJ, van de Wetering M, Barker N, Stange DE, et al Single Lgr5 stem cells build crypt-villus structures in vitro without a mesenchymal niche. Nature. 2009;459: 262–265. 10.1038/nature07935 19329995

[28] Schuijers J, Clevers H. Adult mammalian stem cells: the role of Wnt, Lgr5 and R-spondins. EMBO J. 2012;31: 2685–2696. 10.1038/emboj.2012.149 22617424 PMC3380219

[29] Surwit RS, Kuhn CM, Cochrane C, McCubbin JA, Feinglos MN. Diet-induced type II diabetes in C57BL/6J mice. Diabetes. 1988;37: 1163–1167. 10.2337/diab.37.9.1163 3044882

[30] Roux PP, Ballif BA, Anjum R, Gygi SP, Blenis J. Tumor-promoting phorbol esters and activated Ras inactivate the tuberous sclerosis tumor suppressor complex via p90 ribosomal S6 kinase. Proc Natl Acad Sci U S A. National Academy of Sciences; 2004;101: 13489–94. 10.1073/pnas.0405659101 15342917 PMC518784

[31] Wauson EM, Zaganjor E, Lee A-Y, Guerra ML, Ghosh AB, Bookout AL, et al The G Protein-Coupled Taste Receptor T1R1/T1R3 Regulates mTORC1 and Autophagy. Mol Cell. Cell Press; 2012;47: 851–862. 10.1016/J.MOLCEL.2012.08.001 22959271 PMC3749915

[32] Morgan-Bathke M, Harris ZI, Arnett DG, Klein RR, Burd R, Ann DK, et al The rapalogue, CCI-779, improves salivary gland function following radiation. PLoS One. 2014; 10.1371/journal.pone.0113183 25437438 PMC4249875

[33] Wirawan E, Vanden Berghe T, Lippens S, Agostinis P, Vandenabeele P. Autophagy: for better or for worse. Cell Res. 2012;22: 43–61. 10.1038/cr.2011.152 21912435 PMC3351915

[34] Perros P, MacFarlane TW, Counsell C, Frier BM. Altered taste sensation in newly-diagnosed NIDDM. Diabetes Care. American Diabetes Association; 1996;19: 768–70. 10.2337/diacare.19.7.768 8799637

[35] Lawson WB, Zeidler A, Rubenstein A. Taste detection and preferences in diabetics and their relatives. Psychosom Med. 1979;41: 219–27. Available: http://www.ncbi.nlm.nih.gov/pubmed/472087 10.1097/00006842-197905000-00005 472087

[36] Gondivkar SM, Indurkar A, Degwekar S, Bhowate R. Evaluation of gustatory function in patients with diabetes mellitus type 2. Oral Surgery, Oral Med Oral Pathol Oral Radiol Endodontology. 2009;108: 876–880. 10.1016/j.tripleo.2009.08.015 19913725

